# Elevated mitochondrial genome variation after 50 generations of radiation exposure in a wild rodent

**DOI:** 10.1111/eva.12475

**Published:** 2017-06-22

**Authors:** Robert J. Baker, Benjamin Dickins, Jeffrey K. Wickliffe, Faisal A. A. Khan, Sergey Gaschak, Kateryna D. Makova, Caleb D. Phillips

**Affiliations:** ^1^ Department of Biological Sciences and Museum Texas Tech University Lubbock TX USA; ^2^ Department of Biology Penn State University University Park PA USA; ^3^ School of Science and Technology Nottingham Trent University Nottingham UK; ^4^ Department of Global Environmental Health Sciences Tulane University New Orleans LA USA; ^5^ Faculty of Resource Science and Technology Universiti Malaysia Sarawak Kota Samarahan Sarawak Malaysia; ^6^ International Radioecology Laboratory Slavutych Kiev Region Ukraine

**Keywords:** bank vole, Chernobyl, chronic exposure, environmental radiation, mitochondrial genome

## Abstract

Currently, the effects of chronic, continuous low dose environmental irradiation on the mitochondrial genome of resident small mammals are unknown. Using the bank vole (*Myodes glareolus*) as a model system, we tested the hypothesis that approximately 50 generations of exposure to the Chernobyl environment has significantly altered genetic diversity of the mitochondrial genome. Using deep sequencing, we compared mitochondrial genomes from 131 individuals from reference sites with radioactive contamination comparable to that present in northern Ukraine before the 26 April 1986 meltdown, to populations where substantial fallout was deposited following the nuclear accident. Population genetic variables revealed significant differences among populations from contaminated and uncontaminated localities. Therefore, we rejected the null hypothesis of no significant genetic effect from 50 generations of exposure to the environment created by the Chernobyl meltdown. Samples from contaminated localities exhibited significantly higher numbers of haplotypes and polymorphic loci, elevated genetic diversity, and a significantly higher average number of substitutions per site across mitochondrial gene regions. Observed genetic variation was dominated by synonymous mutations, which may indicate a history of purify selection against nonsynonymous or insertion/deletion mutations. These significant differences were not attributable to sample size artifacts. The observed increase in mitochondrial genomic diversity in voles from radioactive sites is consistent with the possibility that chronic, continuous irradiation resulting from the Chernobyl disaster has produced an accelerated mutation rate in this species over the last 25 years. Our results, being the first to demonstrate this phenomenon in a wild mammalian species, are important for understanding genetic consequences of exposure to low‐dose radiation sources.

## 1 INTRODUCTION

It is well documented that high doses of acute radiation cause mutations with frequent negative genetic and health consequences (Hallahan, Spriggs, Beckett, Kufe, & Weichselbaum, 1989; Hong et al., 1995; Little, Nagasawa, Pfenning, & Vetrovs, 1997; Morgan, Day, Kaplan, McGhee, & Limoli, 1996; Müller et al., 1996; Tucker, Cofield, Matsumoto, Ramsey, & Freeman, 2005; Ward, 1995). What is not known is whether a chronic sublethal dose over a defined number of generations alters the genome, including the mitochondrial genome, of species living in an environment with elevated levels of radioactivity (Premi, Srivastava, Chandy, & Ali, 2009; Wickliffe, Chesser, Rodgers, & Baker, 2002; Wickliffe et al., 2006). Nonetheless, one of the greatest human fears is that exposure to radiation may result in genetic mutations that will result in birth defects and compromised health in future generations. Because environments which have suffered nuclear disasters exhibit significant levels of radiation for hundreds of years, quantifying any genetic effects of long‐term low‐dose exposure is highly relevant to both human and environmental health. The environment created by the Chernobyl meltdown on 26 April 1986, is inhabited by animals and plants offering an opportunity to test for the consequences and evolutionary implications of multigenerational exposure to substantial chronic radiation by comparing populations at the most contaminated localities to those from nearby uncontaminated localities (Bickham & Smolen, 1994).

The bank vole (*Myodes glareolus)* is a common rodent (Baker et al., 1996) in the most radioactive sites adjacent to Chernobyl, the Red Forest and Glyboke Lake. This species experiences both high external and internal doses of radiation in these habitats (Table 1; Chesser et al., 2000, 2001). For several years after the explosion, bank voles experienced annual doses that if acutely delivered would exceed the LD50_30_ (the dose expected to cause death of 50% of an exposed population within 30 days) reported for *Myodes* (10 Gy, Dunaway, Lewis, Story, Payne, & Inglis, 1967; Buech, 1971). As reported by Chesser et al. (2000, 2001), during 1994–1996 bank voles in the Red Forest directly absorbed doses from ^137^cesium and ^90^strontium ranging from 0.44 to 60 mGy per day. This level of radiation is equivalent to 4–600 chest X‐rays, or up to eight chest CT scans per day.

**Table 1:** Samples sizes, coordinates and doses across localities and time points

Locality	*N*, 1998	*N*, 2011	Coordinates	Av, absorbed dose (2011; microGy/day)
Contaminated				
Red Forest	20	17	51.28302, 30.06140	6,670
Glyboke Lake	15	3	51.44603, 30.06645	1,835
Uncontaminated				
Nedanchychy	11	12	51.49533, 30.64636	3.8
Nezamozhnya	12	12	51.58829, 30.85155	2.6
Oranoe	15	14	51.04747, 30.16034	6.9

Based on isotopic composition and decay rates, absorbed doses in the Red Forest and Glyboke Lake regions were substantial enough to cause local extinctions and subsequent infertility in the months and years immediately following the disaster. From the mid‐ to late‐1990's, absorbed doses remained high in comparison with other radioactive environments but below those that cause apparent impacts on fertility and fecundity (Chesser et al., 2000, 2001). Thus, animals as part of this study collected at these contaminated sites are likely the result of reproduction occurring after decay to below lethal levels. The likelihood of detecting genetic effects of radiation is greatest in the mitochondrial genome because DNA repair mechanisms regulating this genome are less complex than those present in the nucleus (Kazak, Reyes, & Holt, 2012), and an increased mitochondrial mutation rate relative to the nucleus is a consistent mammalian characteristic. For example, nucleotide excision repair is thought to be absent from mitochondria, while it is not entirely clear if mismatch repair is present (Shaughnessy et al., 2014). Double‐strand break repair (DSBR) is widely held to be deficient in mitochondria in terms of “classical” mechanisms such as nonhomologous end‐joining (NHEJ) although recent evidence indicates that DSBR through other processes such as microhomology‐mediated alternative NHEJ may actually be a robust mechanism for repairing DSBs in mtDNA (Shaughnessy et al., 2014). On the other hand, base excision repair is active in mitochondria, and the BER pathway is largely responsible for correcting oxidative base lesions (Shaughnessy et al., 2014). In this study we sequenced mitochondrial genomes of 131 individual bank voles collected in 1998 and 2011 from the most radioactive sites and from reference sites. We compared population genetic and molecular evolutionary characteristics of localities and time points to test for differences in mitochondrial genomes which may be a function of multigenerational exposure to chronic radiation.

## 2 MATERIALS AND METHODS

Sampling strategy included two time points and five localities sampled at each time point (Table 1). Localities consisted of two contaminated localities and three uncontaminated localities. Average sample size per locality‐time point was 13, and total sample size was 131 individuals. Total genomic DNAs were isolated from muscle tissue using DNeasy Blood and Tissue Kits (Qiagen, Valencia, CA), which had been preserved in liquid nitrogen immediately after sacrifice, and subsequently archived at −80°C at the Genetic Resources Collection, Museum of Texas Tech University. DNA extraction followed the manufacturer's protocol for isolating DNA from animal tissues. Nuclear DNA integrity was verified using 1% agarose gel electrophoresis and comparing DNA mass distributions to 1‐kilobase DNA ladder (New England BioLabs, Ipswich, MA). Samples were considered “high quality” when the high molecular weight band was equal to, or larger than, the 10 kilobase marker. All samples used in this study passed this criterion. Approximately 1 μg of DNA for each individual was prepared into individual barcode shotgun sequencing libraries using Illumina TruSeq DNA Sample Preparation v2 kits (Illumina, San Diego, CA) and shearing with a Covaris S‐Series instrument (Covaris, Woburn, MA) for 60 s to produce median insert sizes of 380 bp. Libraries were fluorometrically quantified using a Qubit instrument (Thermo Fisher Scientific, Waltham, MA) and titrated to 1/12 lane loadings for 2 × 100 paired‐end multiplexed sequencing on a HiSeq 2000 instrument. Overall sequencing output was approximately 1.9 Tb (~14.5 Gb per individual), 87% of which was retained after quality filtering (Lohse et al., 2012) in which trailing nucleotides were trimmed when quality dropped below a phred‐scaled quality score of 30, and intervals were clipped and excised when average quality dropped below a score of 30 in a 5 bp sliding window. Details of quality filtering results are available in Table S1.

A pilot analysis of 11 individuals distributed across sampling localities was initially used to generate a reference *M. glareolus* mitochondrial genome. Reads for these individuals were aligned to the closest phylogenetic relative with a published mitochondrial genome available at the time of analysis, *Eothenomys chinensis* (GenBank accession number NC_01357.1; Yang et al., 2012), using Bowtie2‐2.1.0 with default settings (Langmead & Salzbergm, 2012). Consensus mitochondrial genomes for each individual were recovered from pileups using Samtools (Li et al., 2009). These genomes were aligned using MUSCLE with default settings (Edgar, 2004), and overall consensus of this alignment was used as the *M. glareolus* reference genome. Next, reads for all samples were aligned to the reference mitochondrial genome as described above, and consensus mitochondrial genomes for each individual were generated as described above. These processing steps resulted in an average depth of coverage per bp across the mitochondrial genome of 3,945 (Table S1). Because Bowtie2 does not map reads that overhang the end of an indexed genome, the leading and trailing 25 bp from each genome were discarded as a quality control measure. Pairwise analysis of variance was used to assess differences in genomic coverage by locality‐time point. The only significant difference was between two uncontaminated localities, Oranoe 2011 and Nezamozhnya 2011 (data not shown); therefore, subsequent analyses did not consider an effect of variance in genomic coverage.

The bioinformatic procedure described above resulted in a final genomic alignment of 16,304 bp for 131 individuals. Ambiguous base calls, which constituted only 0.0015% of the final alignment matrix, were coded as unknown characters. Overall and gene‐specific best‐fit models of molecular evolution were determined following the akaike information criterion implemented in jModelTest2 (Darriba, Taboada, Doallo, & Posada, 2012). Patterns of variation for each gene region were initially described by substitutions per site and frequencies of synonymous and nonsynonymous changes. SLAC (Kosakovsky Pond & Frost, 2005) and MEME (Delport, Poon, Frost, & Kosakovsky Pond, 2010) analyses were performed to estimate mean nonsynonymous‐to‐synonymous rate ratio (ω = d*N*/d*S*) for each gene region and to identify codons exhibiting patterns of variation indicative of positive, negative or episodic directional selection. The distribution of substitutions per site for each gene region across locality‐time points was assessed using *F* tests for equality of variances and Shapiro–Wilk's tests for normality (Shapiro & Wilk, 1965). Following the results of these tests, distributions were compared using either two‐tailed Student's *t* tests or Mann–Whitney *U* tests. Genomic alignments for all individuals are available in Table S2.

A series of population genetic statistics were calculated to compare levels of genetic diversity among locality‐time points. The statistics calculated were (i) the number of haplotypes serving as a basic measure of genome diversity; (ii) the number of polymorphic sites serving as a basic descriptor of nucleotide variability; (iii) both (i) and (ii) divided by locality‐time point sample sizes to control for sample size; (iv) gene diversity (Nei, 1987), as the probability that two randomly drawn haplotypes from a locality‐time point are different; (v) π, average pairwise genetic distance between individuals within a locality‐time point; and (vi) Tajima's *D*, typically an excess of low frequency alleles would indicate demographic expansion or diversifying selection, but in this case would also be compatible with generation of genetic variation by ionizing radiation. To identify statistically significant differences between groups, two‐tailed Student's *t* tests and Mann–Whitney *U* tests were performed for the number of haplotypes, the number of polymorphic sites and gene diversity, and each statistic being corrected for locality‐specific sample size differences. For statistical testing, each locality‐time point was treated as a statistical testing unit, which were categorized as either contaminated or uncontaminated. Glyboke Lake 2011, locality‐time point was removed from this analysis due to small sample size (*n* = 3). Genetic distances between time points within each locality were calculated, ranked and subsequently evaluated by power analyses.

To further identify any effects on locality‐specific statistics by sampling error the most common haplotype in the overall population (15.3% of the sample) was removed from the data set, and population genetic statistics mentioned above were recalculated for each locality‐time point. In addition, because sample size was the largest for Red Forest 1998, the total sample at this locality‐time point (*n* = 20) was randomly subsampled to the average locality‐time point sample size (*n* = 13) through 100 iterations, and number of polymorphic sites and gene diversity estimates were recalculated. Following Shapiro–Wilk's tests for normality, permuted subsampling distributions were compared to uncontaminated localities using Mann–Whitney *U* tests.

## 3 RESULTS

A total of 495 variable positions were identified across the mitochondrial genome alignment of 16,304 bp (Table 2, Table S2). In order to quantify the distribution of variation across gene regions the partitioning of observed variation was tabulated, resulting in five to 70 variable sites identified per gene region, with a range of average sequence variability of 0.59%–4.79%. No insertion‐deletion mutations (indels) were identified through genomic comparisons. This is not unexpected given the lack of introns in mitochondrial DNA (for which strong selection against frame‐shifting indels is expected). Additionally, the levels of radiation and absorbed doses experienced by *M. glareolus* in the Chernobyl environment are not expected to induce clustered oxidative lesions that tend to result in strand breaks and deletion mutations, but rather point mutations as has been observed in some previous studies examining low‐dose radiation exposures including chronic exposures (Forster, Forster, Lutz‐Bonengel, Wollkomm, & Brinkmann, 2002; Schwartz, Jordan, Sun, Ma, & Hsie, 2000). A predominance of synonymous substitutions was reflected in SLAC estimated mean ω for gene regions which ranged from 0 to 0.24 (Table 2). No evidence of positive, negative or episodic directional selection was identified through any analysis (*p *>* *.05). Comparison of average substitutions per site estimated for contaminated and uncontaminated localities across the entire mitochondrial genome resulted in significantly greater substitutions per site at contaminated localities as compared to uncontaminated localities (*t* = 4.08, two‐tailed *p *<* *.05, Effect size (*d*) = 2.64). Similar comparisons for each gene revealed apparently higher mean substitutions per site for contaminated localities for all gene regions except ND3 and Cytb. Subsequent statistical testing identified COI, COIII, ND1, ND4, ND5 and D‐Loop as having significantly greater substitutions per site at contaminated localities as compared to uncontaminated localities (Table S3). Overall, mitochondrial genomic comparisons as well as comparisons at the gene level report patterns of greater genetic variation among contaminated localities as compared to uncontaminated localities.

**Table 2:** Distribution of variation across mitochondrial genes/regions

Gene	Size (bp)	Variable sites	Divergence	Synonymous	Nonsynonymous	ω
tRNAs	1,450	25	1.72%	—	—	—
12S	948	16	1.69%	—	—	—
16S	1,571	22	1.40%	—	—	—
ND1	960	46	4.79%	41	5	0.07
ND2	1,035	45	4.35%	36	9	0.08
COI	1,545	49	3.17%	47	2	0.01
COII	684	17	2.49%	17	0	0
ATP8	204	5	2.45%	4	1	0.12
ATP6	681	22	3.23%	16	6	0.15
COIII	784	33	4.21%	27	6	0.08
ND3	348	11	3.16%	8	3	0.24
ND4L	297	8	2.69%	6	2	0.15
ND4	1,378	60	4.35%	50	10	0.08
ND5	1,812	70	0.59%	50	20	0.15
ND6	525	11	2.10%	9	2	0.08
CytB	1,144	23	2.01%	20	3	0.08
D‐Loop	988	32	3.24%	—	—	—
Total (T)/Average (A)	16,354 (T)	495 (T)	2.8% (A)	331 (T)	69 (A)	0.10 (A)

To further quantify differences in genetic variation across localities, a series of population genetic measures and statistical tests were evaluated (Table 3). Consistent with the hypothesis of a genetic effect associated with chronic exposure to radiation, for all statistics considered, including number of haplotypes, number of polymorphic sites, gene diversity, π, and Tajima's *D,* contaminated localities retained higher values than uncontaminated localities. Correcting for locality‐time point sample sizes, both Student's two‐tailed *t* tests and Mann–Whitney *U* tests indicated significantly greater numbers of haplotypes (*t* = 2.97, *p *<* *.02, *U* = 0, *p *<* *.03, *d *=* *2.46), polymorphic sites (*t* = 2.36, *p *<* *.05, *U* = 0, *p *<* *.03, *d *=* *1.93) and genetic diversity (*t* = 2.79, *p *<* *.03, *U* = 0, *p *<* *.03, *d *=* *2.35) at contaminated localities as compared to uncontaminated localities, while no statistically significant differences for any of these population genetic measures were found when years were compared (Table 4). To assess genetic effects of radiation exposure accumulating between sampling time points, the average number of nucleotide differences between time points within each sampling locality was calculated. Ranking these identified the largest values for comparisons between contaminated localities (Table S4). Although the number of localities precluded statistical testing for a genetic effect between time points within localities, a power analysis incorporating a sample size imbalance of 1.5 (equal to that of the current data) indicated that the inclusion of four contaminated localities and six uncontaminated localities would be required to obtain significance at *p *=* *.05 and power (1−error probability) = 0.8.

**Table 3:** Population genetic summary statistics for each locality‐time point

Locality/Year	Haplotypes	Polymorphic Sites	Gene Diversity	π	Tajima's *D*
Contaminated					
Red Forest 1998	***13*** (0.65)	***216** (10.8)*	0.94 (0.03)	44.29 (20.05)	−1.14 (0.11)
Red Forest 2011	12 (0.71)	175 (10.29)	***0.96 (0.03)***	37.23 (17.05)	−***1.21 (0.10)***
Glyboke Lake 1998	11 ***(0.73)***	174 **(11.6)**	0.95 (0.04)	***44.88 (20.63)***	−0.72 (0.25)
Glyboke Lake 2011	—	—	—	—	—
Uncontaminated					
Nedanchychy 1998	4 (0.36)	85 (7.73)	0.60 (0.15)	24.98 (11.9)	−0.68 (0.25)
Nedanchychy 2011	3 (0.25)	59 (4.92)	0.73 (0.06)	28.71 (13.53)	2.14 (~1)
Nezamozhnya 1998	7 (0.58)	116 (9.67)	0.86 (0.08)	39.02 (18.26)	0.06 (0.59)
Nezamozhnya 2011	6 (0.5)	120 (10)	0.76 (0.12)	36.54 (17.13)	−0.39 (0.37)
Oranoe 1998	9 (0.6)	136 (9.07)	0.90 (0.05)	36.28 (16.74)	−0.6 (0.33)
Oranoe 2011	6 (0.43)	110 (7.86)	0.79 (0.09)	31.15 (14.48)	−0.46 (0.34)
Average % increase	206 (154)	181 (133)	(123)	(129)	

**Figure 1:**
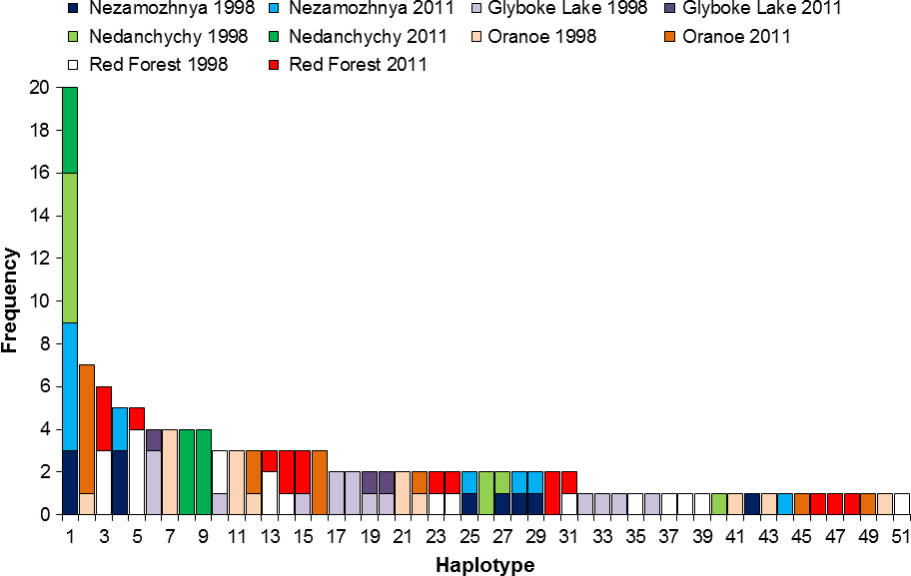
Frequency distributions of observed mitochondrial genome haplotypes across locality‐time points

Values in parentheses are number of haplotypes divided by locality‐time point sample size, number of polymorphic sites divided by sample size, gene diversity standard error, pairwise differences standard error and Tajima's *D p*‐value. Values are not shown for Glyboke Lake 2011 (*n* = 3). Largest values for each statistic (most negative for Tajima's *D*) are bold and italicized. Average % increase for contaminated sites is also provided. See Figure 1 for haplotype frequency distributions.

**Table 4:** Test statistics of major comparisons based on values corrected by locality‐specific sample sizes

Sample comparison	Contrast	Student's *t* test statistic (*p*‐value)	Mann–Whitney *U* test
Contaminated vs. Uncontaminated			
Haplotypes	0.70 vs. 0.45	**2.95 (<.02)**	**<0.03**
Polymorphic sites	10.9 vs. 8.2	**2.37 (<.05)**	**<0.03**
Genetic diversity	0.95 vs. 0.77	**2.73 (<.03)**	**<0.03**
1998 vs. 2011			
Haplotypes	0.59 vs.0.47	1.06 (<.33)	<0.42
Polymorphic sites	9.8 vs. 8.3	1.13 (<.47)	<0.56
Genetic diversity	0.85 vs. 0.81	0.52 (<.63)	<0.74

Glyboke Lake 2011 (*n* = 3) was removed for these comparisons. Significant comparisons are in bold.

**Figure 2:**
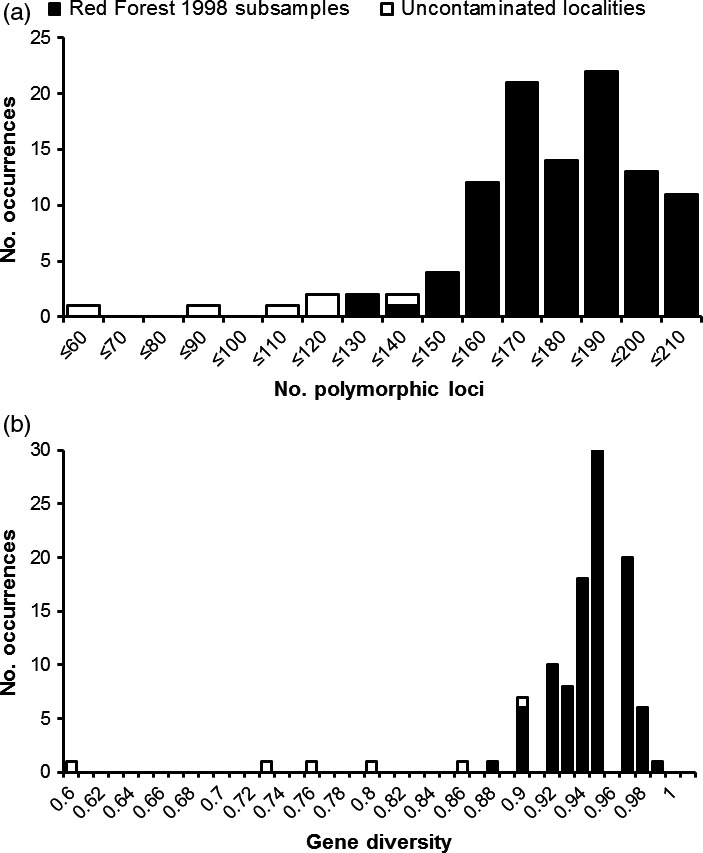
Frequency distributions for (a) number of polymorphic sites and (b) gene diversity, estimated from 100 permutated subsamples of Red Forest 1998 to the study‐wide locality‐time point average sample size (*n* = 13); uncontaminated locality‐time point values are included for comparison

To detect effects stemming from the spatial distribution of genetic variation on locality‐time point sampling error, the most common haplotype (Figure 1) was removed, and population genetic statistics were re‐evaluated. These tests resulted in similar statistical results to those obtained from analysis of the full data set (Table S5). The locality‐time point with the largest sample size (Red Forest 1998) was further evaluated as an additional measure to estimate any influence of locality‐time point sample sizes on statistical results. A permuted subsampling of this locality‐time point's sample size (*n* = 20) to that of the average locality‐time point sample size (*n* = 13) resulted in a distribution of polymorphic sites with a mean and standard deviation of 176 and 18, respectively, whereas mean and standard deviation of uncontaminated sites were 104 and 25, respectively. These distributions were found to be significantly different from each other (Mann–Whitney *U* = 2, *p *<* *.01; Figure 2a). Similarly, these permuted data sets yielded gene diversity estimates within contaminated sites with mean and standard deviation of 0.94 and 0.02, respectively, whereas mean and standard deviation of uncontaminated sites were 0.78 and 0.1, respectively. These distributions were also significantly different from each other (*U* = 7, *p *<* *.01; Figure 2b).

## 4 DISCUSSION

While evolutionary impacts have been suggested in other vertebrate and invertebrate species exposed to physical and chemical toxicants, the mitochondrial genome comparisons in this study are the first to detect a statistically significant difference in the genetic diversity of a native, resident mammalian species encountering multigenerational chronic exposure to radiation in any contaminated environment (Matson et al., 2006; Møller, ErritzøE, Karadas, & Mousseau, 2010; Møller & Mousseau, 2006). Patterns indicate that multigenerational low‐dose radiation exposure has increased the mitochondrial mutation rate in this species in contaminated localities examined thus far. Because populations were most likely extirpated in the localities with the highest contamination immediately following the Chernobyl nuclear meltdown, bank vole populations currently inhabiting these areas are the result of subsequent recolonization. Population genetic expectations are that founded populations will consist of a subset of diversity found in adjacent areas (Mayr, 1942), and an assumption of our study is that populations inhabiting contaminated regions were founded by populations from adjacent uncontaminated regions. Although it is not possible to discern variation introduced by local immigration and that originating from radiation induction, levels of diversity in contaminated localities are greater than in uncontaminated localities, and these observations are inconsistent with a source‐sink scenario.

In spite of the inferred genetic effect of chronic low‐dose radiation exposure, comparison of population sizes and health status (Baker & Chesser, 2000; Baker et al., 1996) is compatible with the hypothesis that any radiation‐induced death‐rate is less than the biological surplus (i.e., more young are born than can survive). Although results are consistent with the hypothesis that an elevated mutation rate is a consequence of living in the radioactive Chernobyl environment, no evidence of any type of selection was inferred. An explanation for this observation is that the relative influence of increased mutation rate and generations of exposure is not sufficient to create an observable signal for selection. Alternatively, the efficiency of natural selection is such that deleterious and advantageous mutations are purged and driven to fixation, respectively, at a rate beyond that resolvable by the current data. The observed preponderance of synonymous mutations supports this scenario. In either case, any cost to populations living in these environments is not obvious. Yet, observing patterns consistent with an accelerated mitochondrial mutation rate suggests an increased likelihood of consequences to genome function and cellular processes. Additionally, results suggest that the genetic variants unique to contaminated localities are inherited by subsequent generations (Figure 1). Our findings are relevant to science and society by informing for the first time genetic outcomes in a wild rodent species experiencing multigenerational low‐dose radiation exposure.

## DATA ARCHIVING STATEMENT

The Dryad repository titled “Data from: Elevated mitochondrial genome variation after 50 generations of radiation exposure in a wild rodent” is now being processed by the curatorial team. The data package has been assigned a unique identifier, called a DOI. This DOI is provisional for now, but may be included in the article manuscript. It will be fully registered with the DOI system when your submission has been approved by Dryad curation staff.

Data package title: Data from: Elevated mitochondrial genome variation after 50 generations of radiation exposure in a wild rodent.

Provisional DOI: https://doi.org/10.5061/dryad.j11s7

Data files: mtDNA gene alignments.
