# Supplementary material for: Elevated mitochondrial genome variation after 50 generations of radiation exposure in a wild rodent
Source: Evol Appl. 2017 Jun 22;10(8):784–91. doi: 10.1111/eva.12475 (PMC5680428; doi:10.1111/eva.12475)
Supplement: Supplementary file 3 [file EVA-10-784-s003.docx]

SI 3. Locality-time point comparisons based on substitutions-per-site for each gene. Means, standard deviations (SD), results of Shapiro-Wilk’s test for normality, and results of F tests for equality of variances are provided. Student’s t-tests assuming equal or unequal variances and Mann-Whitney U test results are provided for corresponding sample comparisons. Significant results for all tests are bolded, italicized, and enclosed in parentheses. Glyboke Lake 2011 (n=3) was removed for these comparisons.

|  | tRNAs | 12S | 16S | | ND1 | | ND2 | | COI | | COII | | | ATP8 | ATP6 | | | COIII | ND3 | | ND4 | | ND4L | | ND5 | | ND6 | | CytB | | D-Loop | | |
| --- | --- | --- | --- | --- | --- | --- | --- | --- | --- | --- | --- | --- | --- | --- | --- | --- | --- | --- | --- | --- | --- | --- | --- | --- | --- | --- | --- | --- | --- | --- | --- | --- | --- |
| Contaminated |  |  |  | |  | |  | |  | |  | | |  |  | | |  |  | |  | |  | |  | |  | |  | |  | | |
| Red Forest 1998 | 0.006 | 0.008 | 0.006 | | 0.018 | | 0.015 | | 0.017 | | 0.016 | | | 0.005 | 0.010 | | | 0.022 | 0.011 | | 0.015 | | 0.013 | | 0.019 | | 0.011 | | 0.007 | | 0.018 | | |
| Red Forest 2011 | 0.005 | 0.005 | 0.004 | | 0.020 | | 0.012 | | 0.014 | | 0.013 | | | 0.010 | 0.013 | | | 0.018 | 0.003 | | 0.012 | | 0.000 | | 0.014 | | 0.006 | | 0.006 | | 0.007 | | |
| Glyboke Lake 1998 | 0.006 | 0.007 | 0.003 | | 0.018 | | 0.017 | | 0.015 | | 0.007 | | | 0.010 | 0.009 | | | 0.015 | 0.011 | | 0.013 | | 0.010 | | 0.014 | | 0.006 | | 0.005 | | 0.011 | | |
| Glyboke Lake 2011 |  |  |  | |  | |  | |  | |  | | |  |  | | |  |  | |  | |  | |  | |  | |  | |  | | |
| Mean | 0.006 | 0.007 | 0.004 | | 0.018 | | 0.015 | | 0.015 | | 0.012 | | | 0.008 | 0.011 | | | 0.018 | 0.009 | | 0.013 | | 0.008 | | 0.016 | | 0.008 | | 0.006 | | 0.012 | | |
| SD | 0.001 | 0.002 | 0.002 | | 0.001 | | 0.003 | | 0.002 | | 0.004 | | | 0.003 | 0.002 | | | 0.003 | 0.005 | | 0.001 | | 0.007 | | 0.003 | | 0.003 | | 0.001 | | 0.006 | | |
| Shapiro-Wilk's test | 0.633 | 0.903 | 0.958 | | 0.851 | | 0.903 | | 1.000 | | 0.903 | | | 0.633 | 0.996 | | | 1.000 | 0.633 | | 0.984 | | 0.842 | | 0.851 | | 0.851 | | 0.984 | | 0.999 | | |
| SW P-value | ***(0.001)*** | 0.445 | 0.766 | | 0.229 | | 0.445 | | 1.000 | | 0.445 | | | ***(0.001)*** | 0.986 | | | 1.000 | ***(0.001)*** | | 0.925 | | 0.203 | | 0.229 | | 0.229 | | 0.925 | | 0.998 | | |
| Uncontaminated |  |  |  | |  | |  | |  | |  | | |  |  | | |  |  | |  | |  | |  | |  | |  | |  | | |
| Nedanchychy 1998 | 0.003 | 0.001 | 0.002 | | 0.003 | | 0.010 | | 0.004 | | 0.003 | | | 0.005 | 0.009 | | | 0.006 | 0.003 | | 0.008 | | 0.000 | | 0.007 | | 0.006 | | 0.007 | | 0.007 | | |
| Nedanchychy 2011 | 0.003 | 0.001 | 0.002 | | 0.003 | | 0.006 | | 0.002 | | 0.001 | | | 0.000 | 0.007 | | | 0.009 | 0.006 | | 0.006 | | 0.000 | | 0.003 | | 0.004 | | 0.005 | | 0.002 | | |
| Nezamozhnya 1998 | 0.003 | 0.004 | 0.004 | | 0.010 | | 0.013 | | 0.006 | | 0.001 | | | 0.000 | 0.007 | | | 0.013 | 0.009 | | 0.013 | | 0.013 | | 0.008 | | 0.002 | | 0.006 | | 0.006 | | |
| Nezamozhnya 2011 | 0.003 | 0.005 | 0.004 | | 0.011 | | 0.014 | | 0.007 | | 0.000 | | | 0.000 | 0.006 | | | 0.013 | 0.009 | | 0.011 | | 0.013 | | 0.008 | | 0.002 | | 0.006 | | 0.007 | | |
| Oranoe 1998 | 0.007 | 0.008 | 0.001 | | 0.014 | | 0.011 | | 0.010 | | 0.003 | | | 0.010 | 0.010 | | | 0.010 | 0.017 | | 0.009 | | 0.003 | | 0.009 | | 0.008 | | 0.009 | | 0.007 | | |
| Oranoe 2011 | 0.003 | 0.007 | 0.002 | | 0.008 | | 0.012 | | 0.008 | | 0.004 | | | 0.010 | 0.009 | | | 0.008 | 0.011 | | 0.007 | | 0.000 | | 0.007 | | 0.006 | | 0.008 | | 0.008 | | |
| Mean | 0.004 | 0.005 | 0.003 | | 0.008 | | 0.011 | | 0.006 | | 0.002 | | | 0.004 | 0.008 | | | 0.010 | 0.009 | | 0.009 | | 0.005 | | 0.007 | | 0.004 | | 0.007 | | 0.006 | | |
| SD | 0.002 | 0.003 | 0.001 | | 0.004 | | 0.003 | | 0.003 | | 0.002 | | | 0.005 | 0.002 | | | 0.003 | 0.005 | | 0.003 | | 0.007 | | 0.002 | | 0.002 | | 0.001 | | 0.002 | | |
| Shapiro-Wilk's test | 0.672 | 0.908 | 0.823 | | 0.890 | | 0.963 | | 0.975 | | 0.959 | | | 0.774 | 0.959 | | | 0.918 | 0.962 | | 0.979 | | 0.734 | | 0.852 | | 0.906 | | 0.958 | | 0.750 | | |
| SW P-value | ***(0.003)*** | 0.424 | 0.094 | | 0.320 | | 0.846 | | 0.922 | | 0.815 | | | ***(0.034)*** | 0.815 | | | 0.492 | 0.833 | | 0.948 | | ***(0.014)*** | | 0.164 | | 0.412 | | 0.802 | | ***(0.020)*** | | |
| F test | 1.095 | 0.808 | 2.729 | | 2.330 | | 2.100 | | 2.638 | | 12.121 | | | 0.948 | 10.530 | | | 6.094 | 1.011 | | 3.944 | | 0.769 | | 5.857 | | 1.534 | | 2.154 | | 7.208 | | |
| F P-value | 0.440 | 0.451 | 0.531 | | 0.144 | | 0.865 | | 0.494 | | ***(0.049)*** | | | 0.552 | 0.431 | | | 0.624 | 0.856 | | 0.501 | | 0.798 | | 0.479 | | 0.449 | | 0.691 | | 0.075 | | |
| 2-tailed t-test |  | 0.637 | 1.056 | | 1.961 | | 0.994 | | 2.679 | | 2.933 | | |  | 0.142 | | | 1.414 |  | | 0.356 | |  | | 2.958 | | 1.654 | | -1.612 | |  | | |
| t-test P-value (equal var.) |  | 0.248 | | 0.096 | | ***(0.007)*** | | 0.095 | | ***(0.002)*** | |  |  | | | 0.068 | ***(0.004)*** | | |  | | ***(0.047)*** | |  | | ***(0.001)*** | | 0.131 | | 0.413 | |  |  |
| t-test P-value (unequal var.) |  |  |  | |  | |  | |  | | 0.053 | | |  |  | | |  |  | |  | |  | |  | |  | |  | |  |  |  |
| Mann-Whitney U | 3 |  |  | |  | |  | |  | |  | | | 4.5 |  | | |  | 8.5 | |  | | 7.5 | |  | |  | |  | | 2.5 | | |
| U P-value | 0.061 |  |  | |  | |  | |  | |  | | | 0.123 |  | | |  | 0.449 | |  | | 0.349 | |  | |  | |  | | ***(0.047)*** | | |
