# Supplementary material for: Elevated mitochondrial genome variation after 50 generations of radiation exposure in a wild rodent
Source: Evol Appl. 2017 Jun 22;10(8):784–91. doi: 10.1111/eva.12475 (PMC5680428; doi:10.1111/eva.12475)
Supplement: Supplementary file 4 [file EVA-10-784-s004.docx]

SI 4. Number of pairwise differences estimated between 1998 and 2011 samples within localities.

| Locality | Number of differences | Standard error |
| --- | --- | --- |
| Glyboke Lake | 34.73 | 3.25 |
| Red Forest | 33.68 | 2.69 |
| Nezamozhnya | 30.47 | 3.20 |
| Oranoe | 30.22 | 4.10 |
| Nedanchychy | 24.48 | 2.78 |
