# Supplementary material for: Elevated mitochondrial genome variation after 50 generations of radiation exposure in a wild rodent
Source: Evol Appl. 2017 Jun 22;10(8):784–91. doi: 10.1111/eva.12475 (PMC5680428; doi:10.1111/eva.12475)
Supplement: Supplementary file 5 [file EVA-10-784-s005.docx]

**SI 5. Population genetic summary statistics for each locality-time point recalculated after removing the most abundant mitochondrial genome haplotype.** Value designations are the same as in Table 4. See Figure 1 for haplotype frequency distributions.

| Locality/Year | Haplotypes | Polymorphic Sites | Gene Diversity | Π | Tajima's *D* |
| --- | --- | --- | --- | --- | --- |
| Contaminated |  |  |  |  |  |
| Red Forest 1998 | ***13*** (0.65) | 175 (8.75) | 0.94 (0.03) | 44.15 (19.99) | -1.14 (0.1) |
| Red Forest 2011 | 12 (0.71) | ***216 (12.71)*** | ***0.96 (0.03)*** | 37.13 (17) | ***-1.21 (0.09)*** |
| Glyboke Lake 1998 | 11 (0.73) | 174 (11.6) | 0.95 (0.04) | ***44.73 (20.56)*** | -0.72 (0.26) |
| Glyboke Lake 2011 | - | - | - | - | - |
| Uncontaminated |  |  |  |  |  |
| Nedanchychy 1998 | 3 (0.75) | 68 (17) | 0.83 (0.22) | 38.28 (21.27) | 0.3 (0.75) |
| Nedanchychy 2011 | 2 (0.25) | 58 (7.25) | 0.57 (0.09) | 33.27 (16.27) | 2.6 (1) |
| Nezamozhnya 1998 | 6 (0.67) | 113 (12.56) | 0.83 (0.13) | 37.84 (18.21) | -0.48 (0.34) |
| Nezamozhnya 2011 | 5 ***(0.83)*** | 98 (16.33) | 0.93 (0.12) | 39.58 (20.14) | -0.52 (0.37) |
| Oranoe 1998 | 9 (0.6) | 136 (9.07) | 0.9 (0.05) | 36.17 (16.7) | -0.6 (0.28) |
| Oranoe 2011 | 6 (0.43) | 110 (7.86) | 0.79 (0.09) | 31.05 (14.44) | -0.46 (0.36) |
